# Supplementary material for: A decision-support framework to optimize border control for global outbreak mitigation
Source: Sci Rep. 2019 Feb 18;9:2216. doi: 10.1038/s41598-019-38665-w (PMC6379393; doi:10.1038/s41598-019-38665-w)
Supplement: Supplementary file 1 — Supplementary Material [file 41598_2019_38665_MOESM1_ESM.pdf]

Supplementary Material

A decision-support framework to optimize border control for global outbreak mitigation

Aleksa Zlojutro[1], David Rey[1], Lauren Gardner[1,2,*]

[1] School of Civil and Environmental Engineering, University of New South Wales (UNSW) Sydney, Sydney, NSW, 2052, Australia
[2] Department of Civil Engineering, Johns Hopkins University, Baltimore, MD 21218, USA

[*]Corresponding Author: l.gardner@unsw.edu.au

A. Illustration of the node control model for paths with stop-overs

Consider a path $k \in \Pi_{ji}$ connecting node j to node i with 2 stop-overs n_1 and n_2, i.e. $k = \{j, n_1, n_2, i\}$. We illustrate the impact of node control on the number of infected individuals which are successfully screened and moved from infectious I to recovered R. Recall that $I_{ji,t}^k$ denotes the compartmental edge flow on path k between j and i at time t. For brevity, we omit the time index t in the remaining of the example. Assume that $I_{ji}^k = 100$ and that the control level at nodes n_1 and n_2 are $x_{n_1} = \frac{1}{4}$ and $x_{n_2} = \frac{1}{5}$, respectively. We consider three cases with different level of control at the destination node i.

From Equation (3c), the number of infectious individuals reaching node i on path k is:

$$I_{ji}^k\left(\prod_{p\in k\setminus\{j\}}(1-x_p)\right) = I_{ji}^k(1-x_{n_1})(1-x_{n_2})(1-x_i) = 100\left(1-\frac{1}{4}\right)\left(1-\frac{1}{5}\right)(1-x_i)$$

From Equation (3d), the number of successfully screened individuals along path k is:

$$I_{ji}^k\left(1-\prod_{p\in k\setminus\{j\}}(1-x_p)\right) = 100\left(1-\left(1-\frac{1}{4}\right)\left(1-\frac{1}{5}\right)(1-x_i)\right)$$

1. $x_i = 0$: the number of infectious individuals reaching node i on path k is: 60 and 40 infectious individuals have been moved to compartment R.
2. $x_i = 1/2$: the number of infectious individuals reaching node i on path k is: 30 and 70 infectious individuals have been moved to compartment R.
3. $x_i = 1$: the number of infectious individuals reaching node i on path k is: 0 and all 100 individuals have been moved to compartment R.

The node control behavior along path k is illustrated in Figure S1 below.

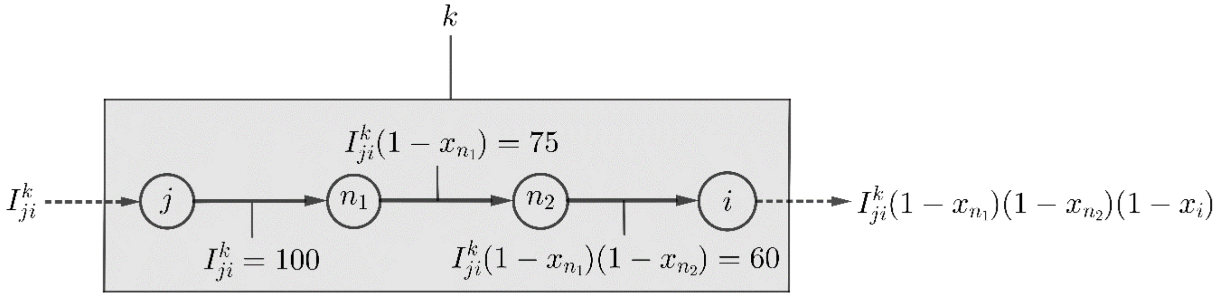

Figure S1. Impact of node control along a path with stop-overs. The number of remaining infectious individuals after each stop-over along the edges of the path. Dashed lines represent initial and final flows.

This example highlights the combined effects of partial node control along a flight route.

B. Base Case Outbreak Dynamics

The U.S. level outbreak dynamics (SIR curves) of the base case is represented in Figure S2 for all three scenarios. For the base case analysis, the hypothetical virus has values of $\alpha = 0$, $\beta = 0.25$, $\gamma = 0.143$ and $\lambda = 1$. The chosen baseline parameters correspond to a disease with a reproductive ratio $R_0 = 1.75$. The outbreak is depicted over the first 300 days, which captures the entire cycle. All three scenarios peak at approximately the same time (t=150 days). Under a do-nothing scenario, the final proportion of the U.S. population infected is around 70%. At t_{obs}=50, the planning time period of focus in this study, only 0.01% of the U.S. population is infected, or around 30 to 35k individuals on average.

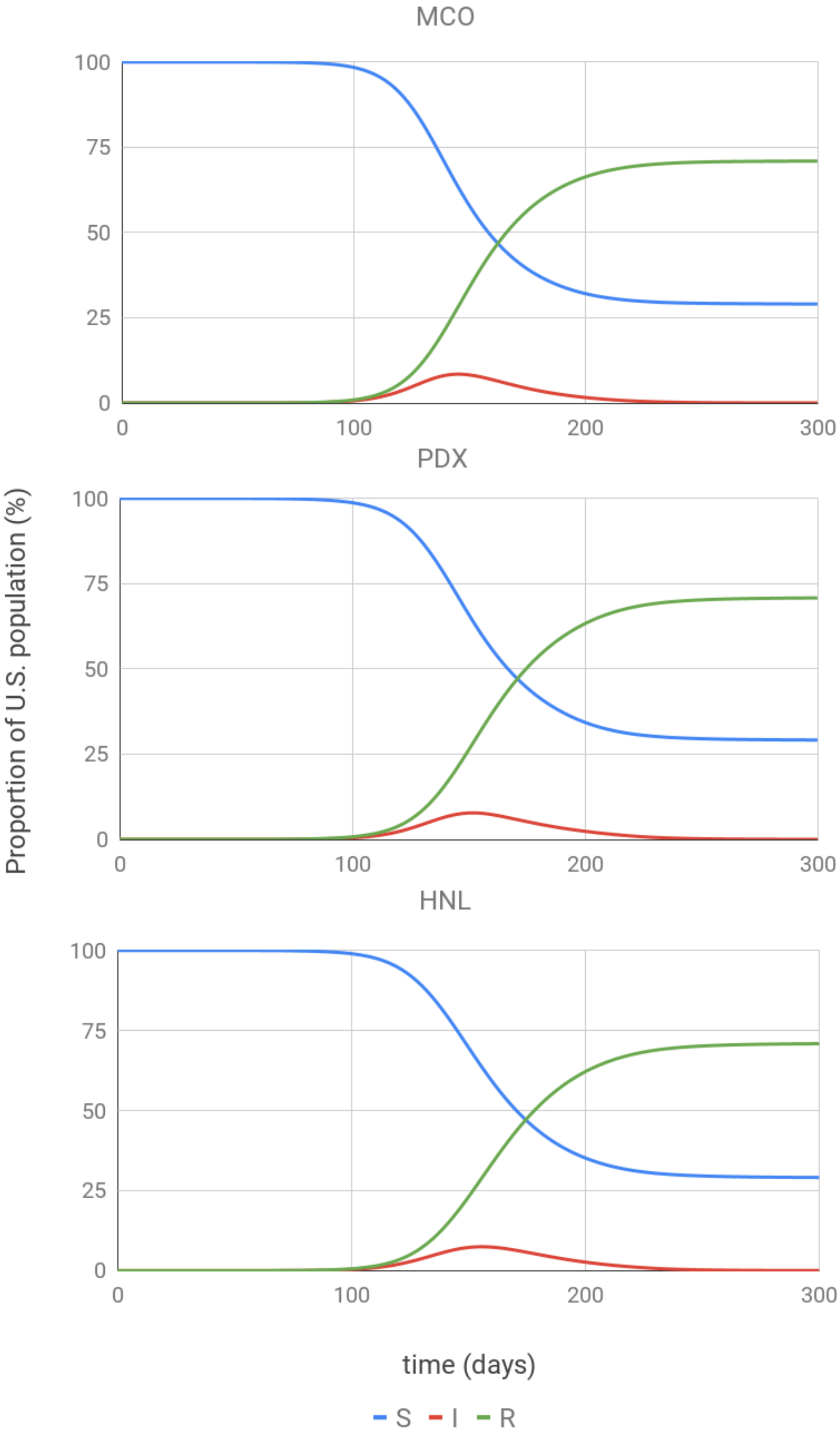

Figure S2. SIR curves for base case scenarios MCO, PDX and HNL. The figure reports the proportion of the U.S. population in each compartmental state (S,I,R) over the course of the outbreak.

C. Sensitivity Analysis

To investigate how the strategies respond to the various parameters and model assumptions, we conduct a range of sensitivity analysis. We explore how changes in the contact rate, the target observation time, *i.e.* t_{obs}, control start time (delayed control), imperfect compliance, and screening outgoing travelers at the source of infection impact the performance of each control strategy. For this sensitivity analysis the set of source cities and base case conditions remain the same as those described in the main document (see Base Case Analysis)

C.1. Impact of the Contact Rate, $\boldsymbol{\beta}$

A significant source of uncertainty in a novel infectious disease is its level of infectiousness, *i.e.*, how fast it will spread. To explore the robustness of our proposed control strategies to the infectivity of a disease, we conduct sensitivity for a range of contact rates. The results for β = 0.2, 0.25 and 0.3 are shown for all scenarios MCO, PDX and HNL, in Figures S3a, S3b and S3c, respectively. These three contact rates (CR in the figures) correspond to a disease with a reproductive ratio $R_0 = 1.4$, 1.75 and 2.1, respectively.

As expected, the number of cases increases with the contact rate for all control strategies. The relative performance of the control strategies compared with the baseline case (corresponding to no control being deployed) increases as the contact rate increases, indicating that the control strategies are more effective for more aggressive outbreaks. Specifically, for the PDX scenario, the EP and MC strategies provide a 16% decrease in cases for β = 0.2 compared with a 24.9% for β = 0.3, relative to the baseline. The gap between the poorest performing strategy, MT, and the best performing strategy also increases with β. Across the range of β, EP and MC consistently perform best. These trends are reflected in scenarios MCO and HNL, with the latter exhibiting a significantly lower volatility in terms of performance compared to the other source city scenarios.

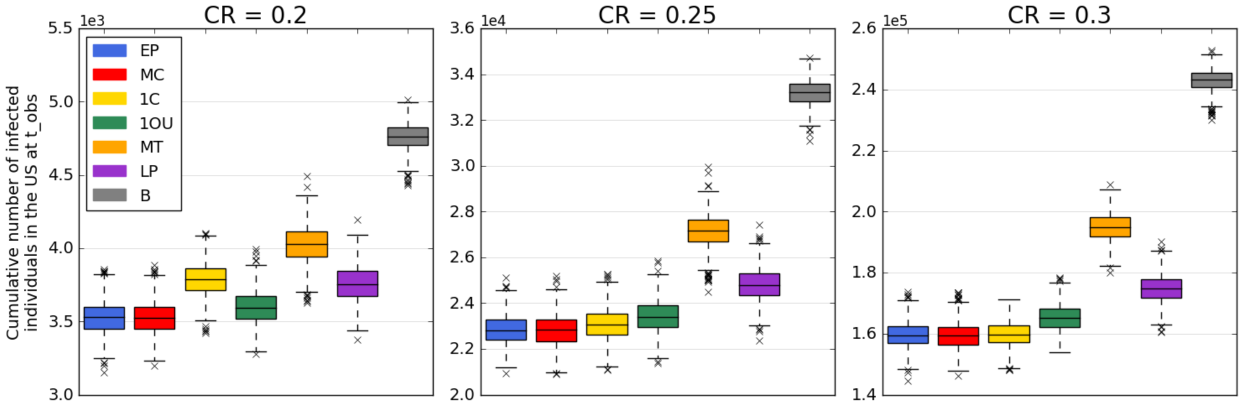

Figure S3a. Impact of the contact rate on the control strategies for the MCO scenario. The figure reports the cumulative number of infected individuals in the U.S. at the observation time $\boldsymbol{t_{obs}} = \mathbf{50}$ days for each control strategy evaluated based on the contact rate (CR), denoted $\boldsymbol{\beta}$ in the formulation. Each boxplot represents the distribution of the criterion measured over 1,000 simulations of the stochastic metapopulation epidemic model under the corresponding control strategy.

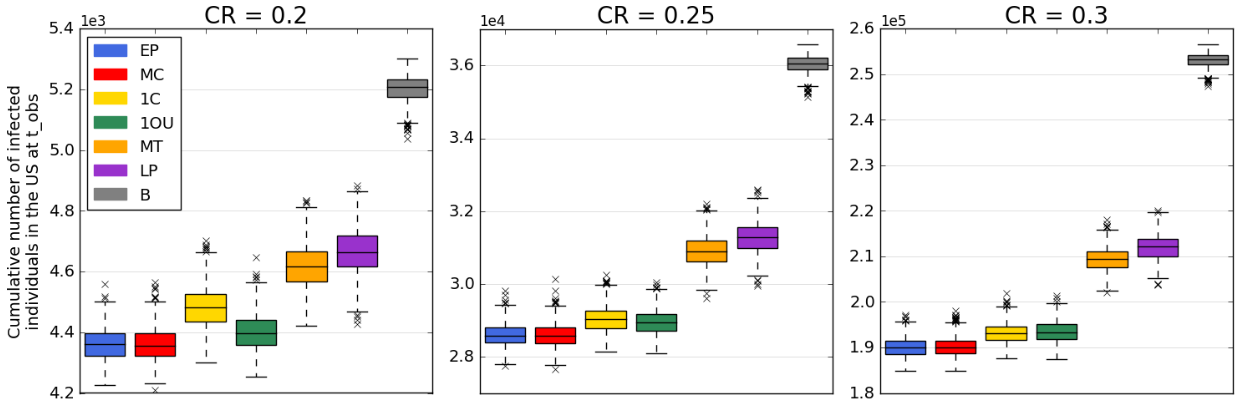

Figure S3b. Impact of the contact rate on the control strategies for the PDX scenario. The figure reports the cumulative number of infected individuals in the U.S. at the observation time $\boldsymbol{t_{obs}} = \mathbf{50}$ days for each control strategy evaluated based on the contact rate (CR), denoted $\boldsymbol{\beta}$ in the formulation. Each boxplot represents the distribution of the criterion measured over 1,000 simulations of the stochastic metapopulation epidemic model under the corresponding control strategy.

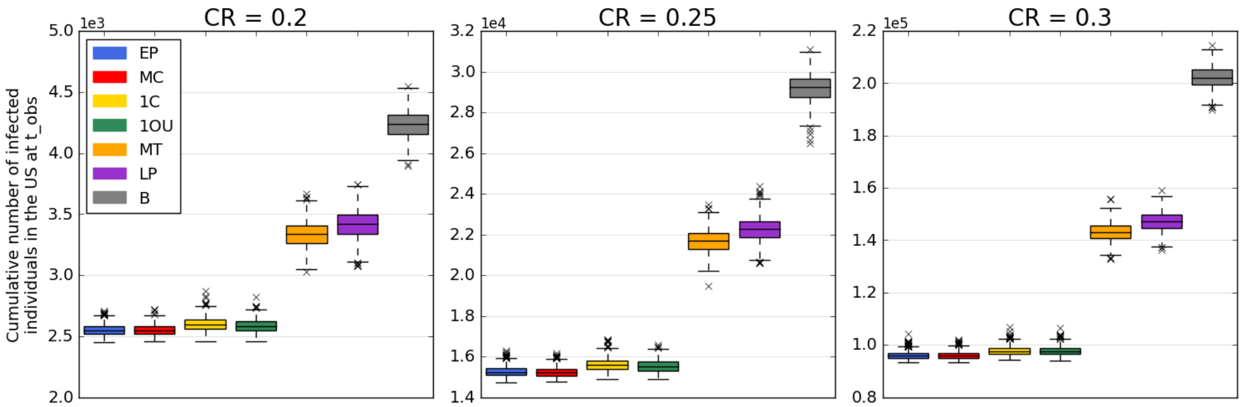

Figure S3c. Impact of the contact rate on the control strategies for the HNL scenario. The figure reports the cumulative number of infected individuals in the U.S. at the observation time $\boldsymbol{t_{obs} = 50}$ days for each control strategy evaluated based on the contact rate (CR), denoted $\boldsymbol{\beta}$ in the formulation. Each boxplot represents the distribution of the criterion measured over 1,000 simulations of the stochastic metapopulation epidemic model under the corresponding control strategy.

C.2. Impact of varying the observation time

The proposed decision-support framework is based on the chosen time of observation, t_{obs}. As previously discussed, the focus of this work is to help guide control decision at the early stages of an outbreak, with the intention of preventing introductions into new cities and regions. Thus, the planning horizon focuses on the first weeks and months after a new disease has been identified. However, the impact of the chosen planning horizon is critical to quantify, as it directly relates to the budget, *e.g.*, a longer planning horizon requires more days of screening, and therefore less airports can be controlled for the same budget. A sensitivity analysis is conducted to measure the impact of t_{obs} when this parameter is varied between 25 and 100 days and its impact on the control strategy performance is assessed. Figures S4a, S4b and S4c provide the results of the sensitivity analysis for scenarios MCO, PDX and HNL, respectively. As t_{obs} increases, the number of cumulatively infected individual grows quickly, highlighting the non-linear growth of outbreaks. The relative impact of the best control strategies, MC and EP, continues to increase with t_{obs}, and critically, they consistently dominate in terms of performance across planning horizons, indicating the strategies are robust. For the MCO scenario, we find that the control strategies perform increasingly similarly when the observation time increases. This is not the case for the PDX scenario wherein increasing t_{obs} magnifies the differences of the control strategies in terms of performance. Finally, the HNL scenario is found to be the most robust to the variation of the observation time and this trend is also reflected more generally for this source city scenario.

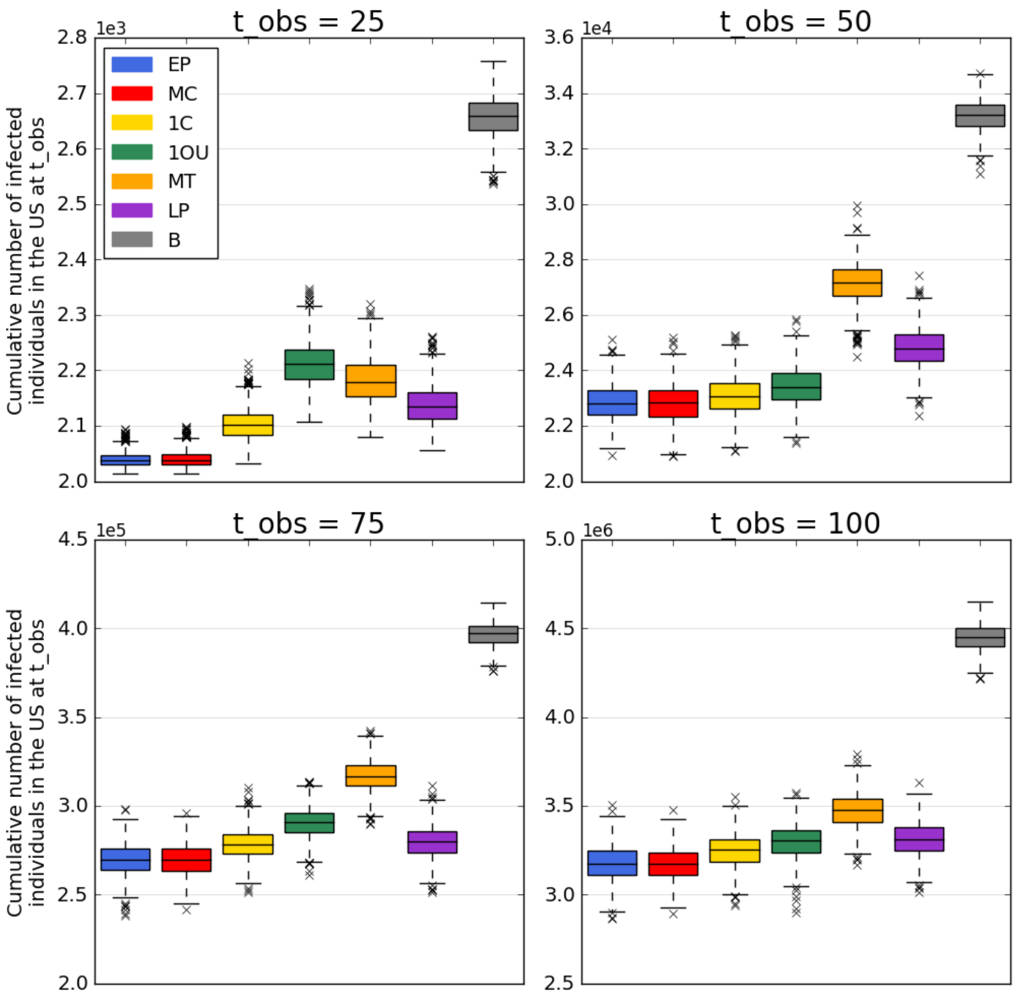

Figure S4a. Impact of changing t_{obs} on the control strategies for the MCO scenario. The figure reports the cumulative number of infected individuals in the U.S. at the observation time $t_{obs} = 50$ days for each control strategy evaluated based on the time of observation t_{obs}. Each boxplot represents the distribution of the criterion measured over 1,000 simulations of the stochastic metapopulation epidemic model under the corresponding control strategy.

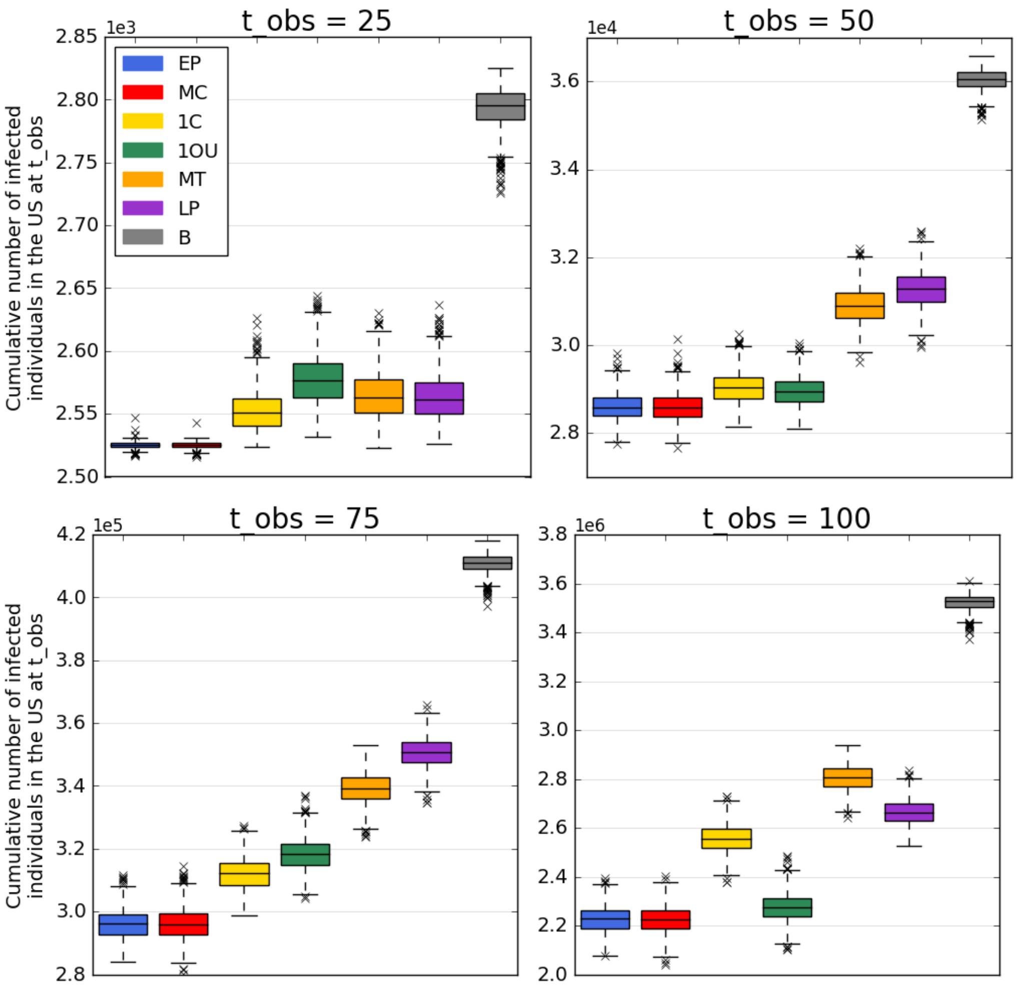

Figure S4b. Impact of changing t_{obs} on the control strategies for the PDX scenario. The figure reports the cumulative number of infected individuals in the U.S. at the observation time $t_{obs} = 50$ days for each control strategy evaluated based on the time of observation t_{obs}. Each boxplot represents the distribution of the criterion measured over 1,000 simulations of the stochastic metapopulation epidemic model under the corresponding control strategy.

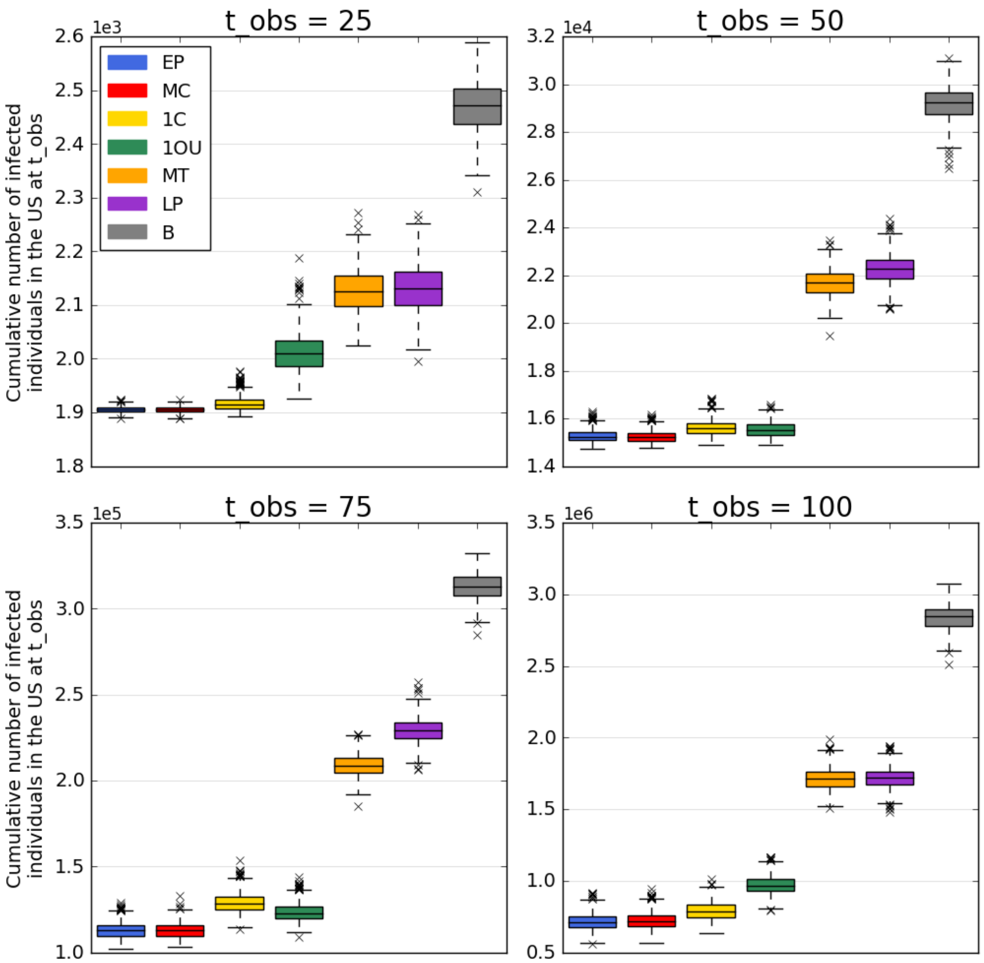

Figure S4c. Impact of changing t_{obs} on the control strategies for the HNL scenario. The figure reports the cumulative number of infected individuals in the U.S. at the observation time $t_{obs} = 50$ days for each control strategy evaluated based on the time of observation t_{obs}. Each boxplot represents the distribution of the criterion measured over 1,000 simulations of the stochastic metapopulation epidemic model under the corresponding control strategy.

C.3. Impact of Delaying Control

As this work focuses on the early stages of an outbreak, the impact of delaying the deployment of control resources can provide key insights on policy implementation and practice. To this effect, we conduct a sensitivity analysis time at which control, *i.e.*, passenger screening is deployed. Specifically, a delay of 0, 7, 14, 21 and 28 days is evaluated – we note that in the base case analysis, control is assumed to start at $t = 0$ whereas in the case study control is assumed to start at $t = 28$ days. In these scenarios the airports control sets for each strategy remain the same as in the base case, with the only difference being the start date of screening. The results of the scenarios are presented in Figure S5. Note: EP performs closely to MC, and is therefore not clearly visible in the figure.

In all base case scenarios MCO, PDX and HNL, delaying the deployment of passenger screening results in more cases. Further, we observe that all control strategies are similarly impacted and their ranking, in terms of their capacity to reduce the number of infected individuals, remain almost always the same (except in scenario HNL, wherein MC/EP and 1OU crossover). The results also highlight that the smarter control strategies (*i.e.*, EP, MC, 1OU and 1C) are more sensitive to delayed implementation, indicating a critical need to implement control as soon as a risk is identified.

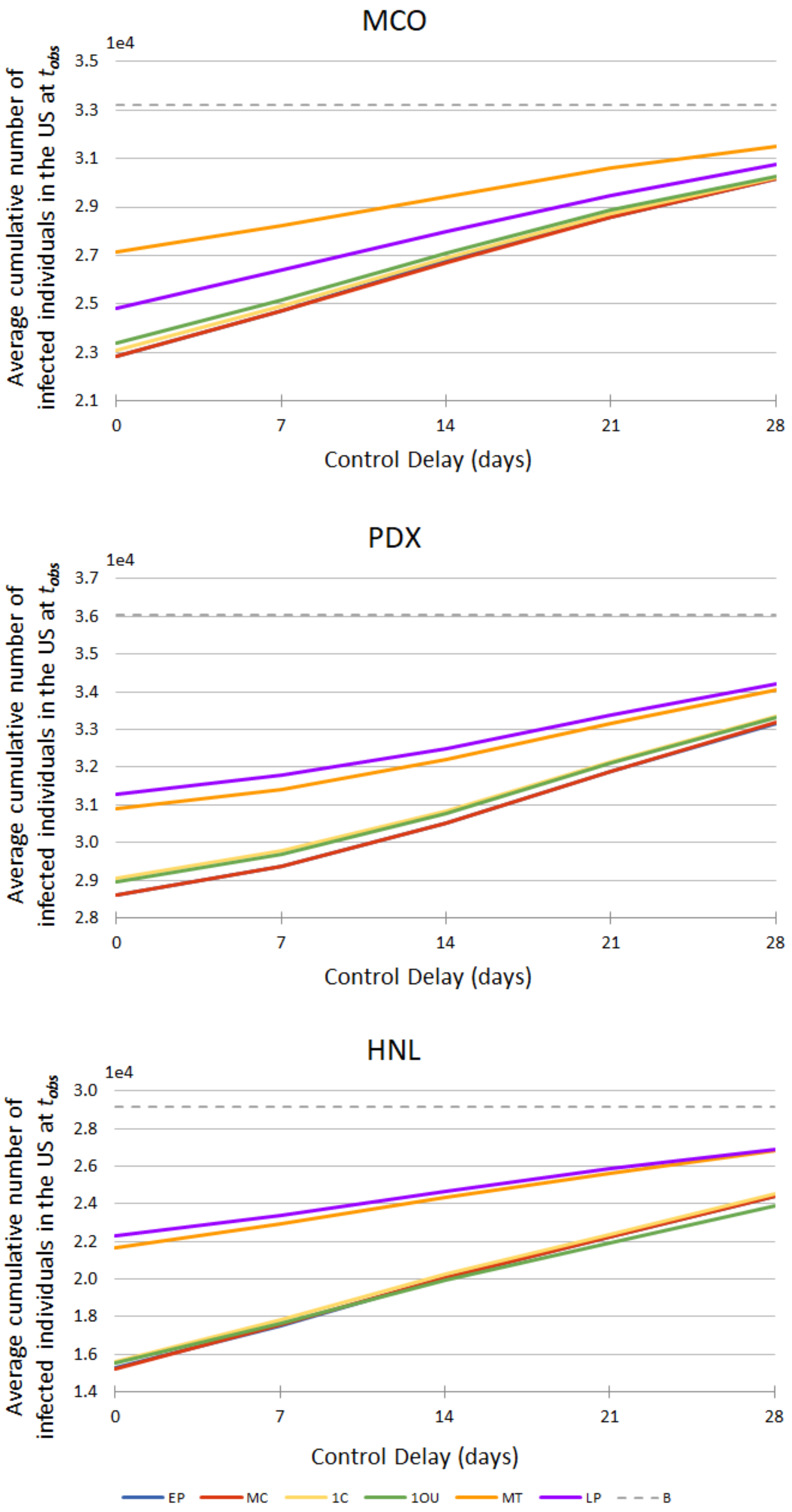

Figure S5. Impact of delayed control the control strategies for base case scenarios MCO, PDX and HNL. The figure reports the average cumulative number of infected individuals in the U.S. for each control strategy evaluated for a varying control start time, *i.e.*, delayed control. Each data point represents the average of the criterion measured over 1,000 simulations of the stochastic metapopulation epidemic model under the corresponding control strategy.

C.4. Impact of Imperfect Compliance

A sensitivity analysis is also conducted to address the modelling assumption of fully successful passenger screening. Indeed, if airport $i \in V$ is fully controlled, *i.e.* $x_i = 1$, our model assumes that all infected individuals are successfully detected in the screening process. In reality, this may not be the case. For example, Auckland International Airport's screening procedure for the 2009 influenza pandemic found that only 6% of infected passengers were detected[1]. This was partially due to the inability of the airport staff to screen all incoming passengers, as well as the limitations of the thermal scanning technology that was used. It is therefore necessary to evaluate the effectiveness of the proposed strategies under imperfect control conditions. To explore the impact of imperfect control, we set upper bounds lower than 1 on the control rate at airports and measure performance in the base case scenarios. The outcome is reported Figure S6. The performance of each control strategy is presented for the cases where the control rate is limited to 0.9 and 0.8. The results are compared with the perfect control scenario. We assume that the same set of airports is controlled (obtained from the base case outcomes) in all simulations.

The results once again highlight the robustness of the control strategies. The increase in infected cases increases as the perfect control assumption is relaxed, however the relative performance and ranking across strategies remains robust to this relaxation. We also report a more apparent increase in cases between full control and 90% control, compared with the difference in 80% and 90% control effectiveness.

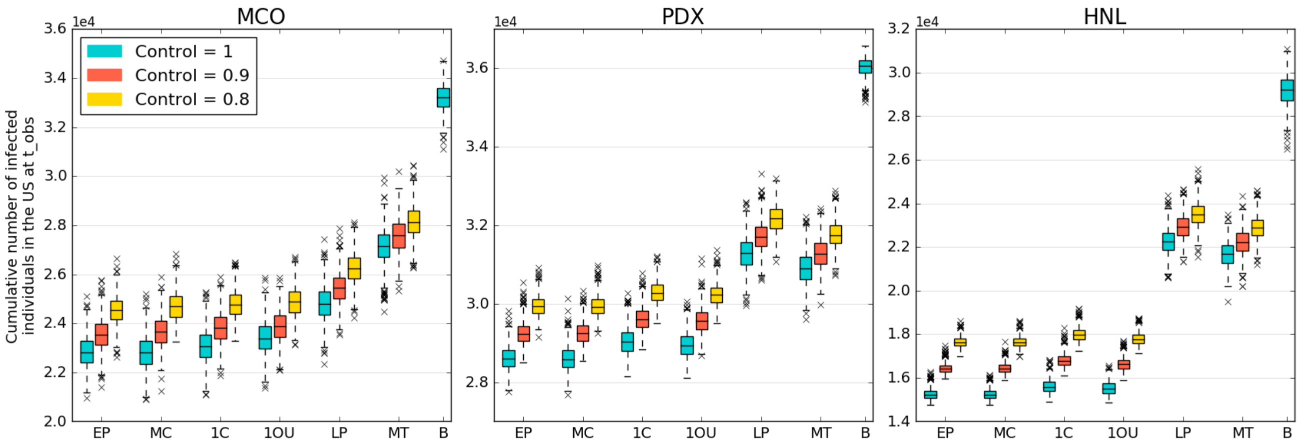

Figure S6. Impact of imperfect compliance on the control strategies for base case scenarios MCO, PDX and HNL. The figure reports the cumulative number of infected individuals in the U.S. for each control strategy evaluated for a varying control start time, *i.e.*, delayed control. Each boxplot represents the distribution of the criterion measured over 1,000 simulations of the stochastic metapopulation epidemic model under the corresponding control strategy.

C.5. Impact of Screening Outgoing Travelers at the Source of Infection

A final sensitivity analysis addresses the issue of outgoing passenger screening at the source of the outbreak. In this work we assumed that screening outgoing passengers at the source city of the outbreak is a trivial decision. Thus, the proposed control strategies focus on which 'other' locations should be prioritized for incoming passenger screening. Furthermore, if outgoing passenger screening is conducted at the outbreak source city, assuming a uniform effectiveness across all intended destinations, the relative performance proposed strategies still applies. This is illustrated in Figure S7, which provides the different control strategy performances and ranking for source screening rates of 0%, 25%, 50%, 75%, 100%. At a 100% outgoing screening rate, no cases will ever leave the source city, thus all strategies perform equally, while the 0% outgoing screening rate corresponds to the base case results. A linear response to source screening is observed for all strategies as the level of source screening is varied. Once again, the strategy rankings remain consistent across scenarios. In all cases, the cost of screening outgoing passengers is excluded from the budget.

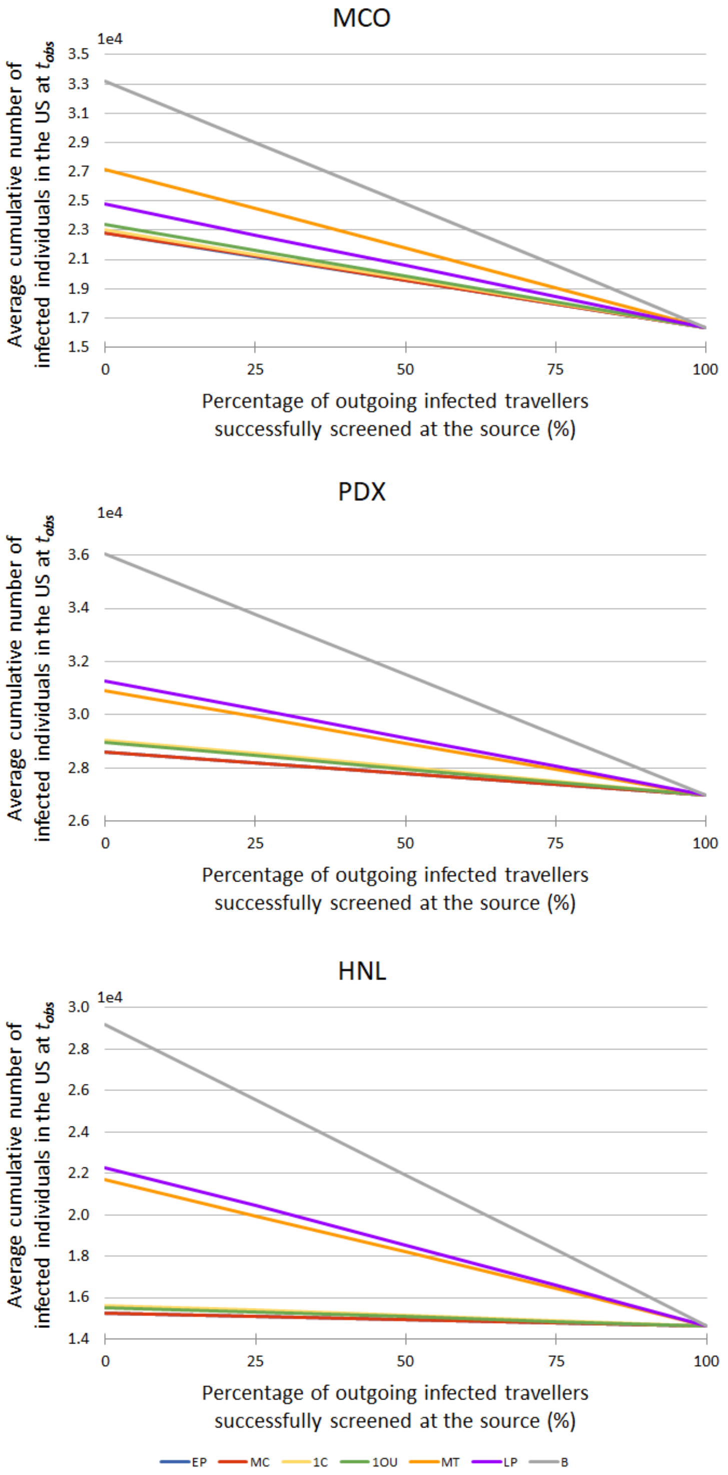

Figure S7. Impact of screening outgoing travelers at the source of infection on the control strategies for base case scenarios MCO, PDX and HNL. The figure reports the cumulative number of infected individuals in the U.S. for each control strategy evaluated for a varying proportion of outgoing infected travelers successfully screened at the source city. Each data point represents the average of the criterion measured over 1,000 simulations of the stochastic metapopulation epidemic model under the corresponding control strategy.

D. H1N1 Case Study Model Calibration

In this section, we provide additional details on the calibration of the proposed stochastic metapopulation epidemic model. The focus of the calibration process was on matching the simulated and reported arrival dates of the first case in a new location, at both the U.S. and global scales. The parameters of the model were set such that the average difference between the reported date of first case[2,3] and the simulated date (when averaged over 1,000 simulations) was minimized. For all simulations the starting date of the outbreak is set as the 5th of February 2009 with 1 infected individual placed into the city of Veracruz, Mexico[4]. Based on the analysis, the best fit disease parameters were found to be $\alpha = 1$, $\beta = 0.475$, $\gamma = 0.25$ and $\lambda = 1$.

Figure S8 compares the observed and simulated date of H1N1 introduction into each of the states in the U.S. for the calibrated model. The model is illustrated to closely capture the trend of first case introductions; critically capturing the gap between the start of the outbreak in Mexico and the time of introduction into California and Texas, after which the outbreak quickly spread to the rest of the country. For the chosen parameters, the average difference between the simulated and observed date of arrival averaged across all 50 states is 7.28 days. The CDC reported dates are later than the dates of initial infection and hence slightly earlier prediction dates by the model are preferable.

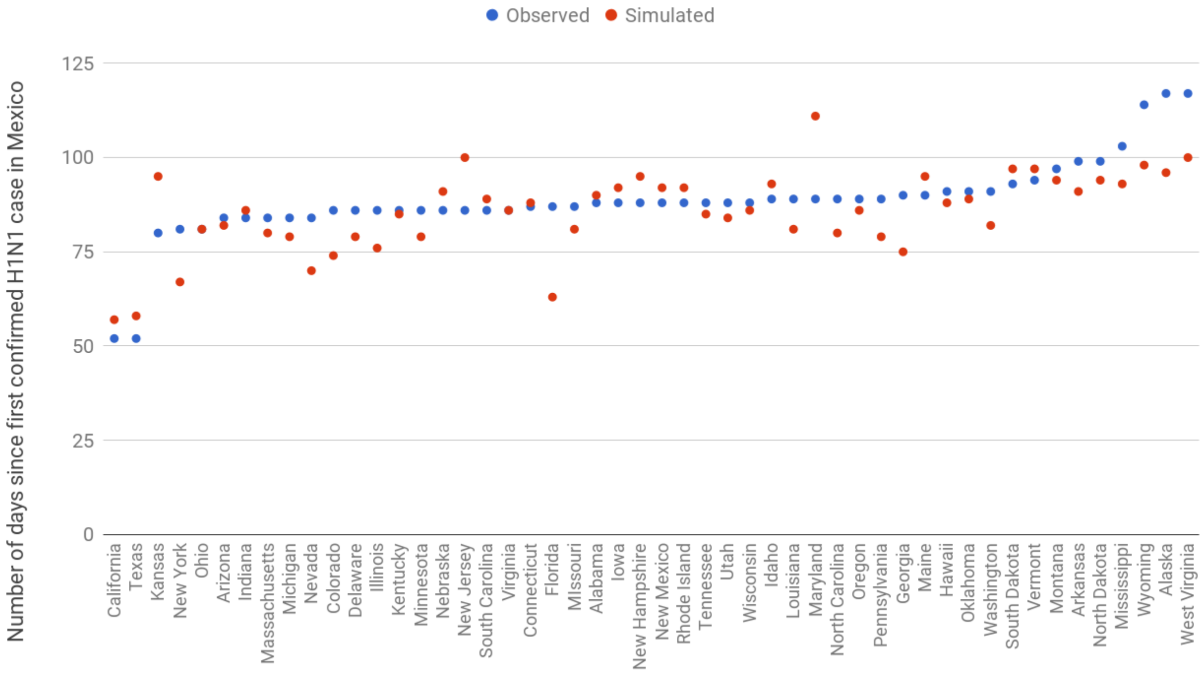

Figure S8. Observed[2] and simulated date of H1N1 introduction into each State in the U.S. The figure shows the observed and simulated date of the introduction of H1N1 in each of the 50 U.S. states sorted by increasing observed dates. The simulated dates are the average over 1,000 simulations of the stochastic metapopulation epidemic model.

A similar analysis was conducted at the global level, to compare the cumulative number of infected countries over time between the simulation and reported data, and illustrated in Figure S9. The difference between the observed[3] and simulated results is minimal; the difference between the cumulative number of infected countries at each date, averaged over the first 100 days, is less than one. The maximum discrepancy during the first 100 days occurs 90 days after the start of the outbreak, at which time the simulation predicts six less countries to be infected.

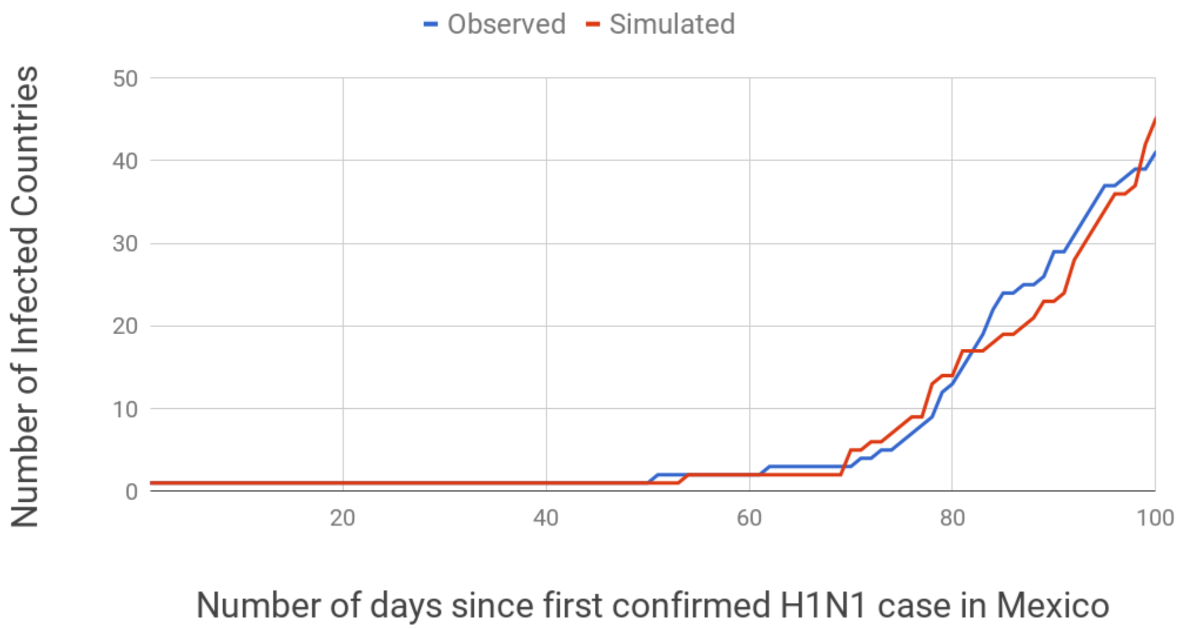

Figure S9. Observed and simulated cumulative number of countries infected over time. The observed curve is based on the date of arrival in country of first confirmed case[3] and the simulated curve is based on the expected date of first arrival averaged over 1,000 simulations of the stochastic metapopulation epidemic model.

To further validate the simulation model at a global level, explicit dates of first case arrivals for 10 countries known to be infected directly from Mexico are compared against the expected date of introduction from the simulation. Table S1[3,5] highlights how closely the simulation captures the observed behavior of the outbreak, with an average difference of 5.4 days across the 10 countries. The observed and simulated arrival dates for all countries except Columbia fall within 8 days.

Table S1. Observed and simulated country infection dates [3,5]. The simulated dates are the average of 1,000 simulations of the stochastic metapopulation epidemic model and rounded to the nearest date.

Country	Observed Arrival Date	Simulated Arrival Date
United States	28/3/2009	30/3/2009
United Kingdom	21/4/2009	23/4/2009
Australia	9/5/2009	9/5/2009
China	2/5/2009	8/5/2009
Canada	8/4/2009	15/4/2009
Spain	22/4/2009	21/4/2009
Germany	28/4/2009	26/4/2009
France	1/5/2009	23/4/2009
Colombia	3/5/2009	15/4/2009
Cuba	25/4/2009	17/4/2009

E. Airport control lists and frequency of control

To provide more details on the behavior of the proposed control strategies we analyse the sets of airports selected for control. For each control strategy, we report the list and the control level of all airports controlled in the base case scenarios MCO, PDX and HNL (note, airports are ordered by decreasing travel volume). We also report the frequency of choosing an airport among the 6 control strategies considered (all airports not listed in the tables are never selected for control and have thus a null frequency). We find that few airports are selected in all 6 control strategies. Even though the strategies have different metrics for ranking airports, EP and MC identify the same lists of airports for control, which explains their similar performance. The two lowest performing control strategies in terms of case numbers, LP and MT, have the most and least number of airports controlled, respectively. For LP, many controlled airports appear to not help in mitigating the spread of the disease, and for MT, the largest airports are always selected for control, which are also the most expensive, and therefore quickly deplete the budget available.

Table S2a. Airport control lists and frequency of airport control across control strategies in the MCO scenario.

EP		MC		1C		1OU		LP		MT		
List	Control	List	Control	List	Control	List	Control	List	Control	MT	Control	Frequency
ATL	1.00	ATL	1.00	ATL	1.00	ATL	1.00	LAX	1.00	ATL	1.00	6
ORD	1.00	ORD	1.00	LAX	0.38	LAX	1.00	ORD	1.00	LAX	1.00	5
DFW	1.00	DFW	1.00	ORD	1.00	ORD	1.00	DFW	1.00	ORD	1.00	4
JFK	1.00	JFK	1.00	DFW	1.00	DFW	1.00	JFK	1.00	DFW	1.00	3
DEN	1.00	DEN	1.00	JFK	1.00	JFK	1.00	SFO	1.00	JFK	1.00	2
CLT	1.00	CLT	1.00	DEN	1.00	DEN	1.00	IAH	1.00	DEN	1.00	1
IAH	1.00	IAH	1.00	CLT	1.00	CLT	1.00	MIA	0.86	SFO	1.00	
MIA	0.06	MIA	0.06	SEA	1.00	IAH	1.00	EWR	1.00	LAS	1.00	
EWR	1.00	EWR	1.00	EWR	1.00	EWR	1.00	BOS	1.00	PHX	1.00	
MSP	1.00	MSP	1.00	MSP	1.00	BOS	1.00	PHL	1.00	CLT	1.00	
BOS	1.00	BOS	1.00	BOS	1.00	DTW	1.00	LGA	1.00	IAH	1.00	
DTW	1.00	DTW	1.00	PHL	1.00	PHL	1.00	BWI	1.00	MIA	1.00	
PHL	1.00	PHL	1.00	LGA	1.00	LGA	1.00	MDW	1.00	SEA	0.77	
LGA	1.00	LGA	1.00	BWI	1.00	BWI	1.00	DCA	1.00			
BWI	1.00	BWI	1.00	MDW	1.00	MDW	0.46	IAD	1.00			
MDW	1.00	MDW	1.00	STL	1.00	SJU	1.00	SAN	1.00			
DCA	1.00	DCA	1.00	SJU	1.00			DAL	1.00			
SJU	1.00	SJU	1.00	IND	1.00			HOU	1.00			
				BDL	1.00			OAK	1.00			
				RHI	1.00			SNA	1.00			
				PAH	1.00			SJC	1.00			
				HIB	1.00			ONT	1.00			
				PKB	1.00			BUR	1.00			
								LGB	1.00			
								HPN	1.00			
								TTN	1.00			
								ILG	1.00			
								CLD	1.00			

Table S2b. Airport control lists and frequency of airport control across control strategies in the PDX scenario.

EP		MC		1C		1OU		LP		MT		
List	Control	List	Control	List	Control	List	Control	List	Control	MT	Control	Frequency
ATL	1.00	ATL	1.00	ATL	1.00	ATL	1.00	LAX	1.00	ATL	1.00	6
LAX	1.00	LAX	1.00	LAX	1.00	LAX	1.00	ORD	1.00	LAX	1.00	5
ORD	1.00	ORD	1.00	ORD	1.00	ORD	1.00	DFW	1.00	ORD	1.00	4
DFW	1.00	DFW	1.00	DFW	1.00	DFW	1.00	JFK	1.00	DFW	1.00	3
JFK	0.45	JFK	0.45	JFK	1.00	JFK	0.77	SFO	1.00	JFK	1.00	2
DEN	1.00	DEN	1.00	DEN	1.00	DEN	1.00	IAH	1.00	DEN	1.00	1
SFO	1.00	SFO	1.00	SFO	1.00	SFO	1.00	MIA	0.86	SFO	1.00	
LAS	1.00	LAS	1.00	LAS	1.00	LAS	1.00	EWR	1.00	LAS	1.00	
PHX	1.00	PHX	1.00	PHX	1.00	PHX	1.00	BOS	1.00	PHX	1.00	
SEA	1.00	SEA	1.00	SEA	1.00	SEA	1.00	PHL	1.00	CLT	1.00	
MSP	1.00	MSP	1.00	MSP	1.00	MSP	1.00	LGA	1.00	IAH	1.00	
SLC	1.00	SLC	1.00	SLC	1.00	SLC	1.00	BWI	1.00	MIA	1.00	
SAN	1.00	SAN	1.00	HNL	1.00	SAN	1.00	MDW	1.00	SEA	0.77	
HNL	1.00	HNL	1.00	OAK	1.00	HNL	1.00	DCA	1.00			
OAK	1.00	OAK	1.00	SJC	1.00	OAK	1.00	IAD	1.00			
SNA	1.00	SNA	1.00	SMF	1.00	SJC	1.00	SAN	1.00			
SJC	1.00	SJC	1.00	GEG	1.00	SMF	1.00	DAL	1.00			
SMF	1.00	SMF	1.00	DBQ	1.00			HOU	1.00			
GEG	1.00	GEG	1.00	JLN	1.00			OAK	1.00			
BOI	1.00	BOI	1.00	PAH	1.00			SNA	1.00			
				EKO	1.00			SJC	1.00			
				BRD	1.00			ONT	1.00			
				HYS	1.00			BUR	1.00			
								LGB	1.00			
								HPN	1.00			
								TTN	1.00			
								ILG	1.00			
								CLD	1.00			

Table S2c. Airport control lists and frequency of airport control across control strategies in the HNL scenario.

EP		MC		1C		1OU		LP		MT		
List	Control	List	Control	List	Control	List	Control	List	Control	MT	Control	Frequency
ATL	1.00	ATL	1.00	ATL	1.00	ATL	1.00	LAX	1.00	ATL	1.00	6
LAX	1.00	LAX	1.00	LAX	1.00	LAX	1.00	ORD	1.00	LAX	1.00	5
ORD	1.00	ORD	1.00	ORD	1.00	ORD	1.00	DFW	1.00	ORD	1.00	4
DFW	1.00	DFW	1.00	DFW	1.00	DFW	1.00	JFK	1.00	DFW	1.00	3
JFK	0.35	JFK	0.35	JFK	0.73	JFK	0.36	SFO	1.00	JFK	1.00	2
DEN	1.00	DEN	1.00	DEN	1.00	DEN	1.00	IAH	1.00	DEN	1.00	1
SFO	1.00	SFO	1.00	SFO	1.00	SFO	1.00	MIA	0.86	SFO	1.00	
LAS	1.00	LAS	1.00	LAS	1.00	LAS	1.00	EWR	1.00	LAS	1.00	
PHX	1.00	PHX	1.00	PHX	1.00	PHX	1.00	BOS	1.00	PHX	1.00	
IAH	1.00	IAH	1.00	IAH	1.00	SEA	1.00	PHL	1.00	CLT	1.00	
SEA	1.00	SEA	1.00	SEA	1.00	MSP	1.00	LGA	1.00	IAH	1.00	
SLC	1.00	SLC	1.00	IAD	1.00	SLC	1.00	BWI	1.00	MIA	1.00	
SAN	1.00	SAN	1.00	SAN	1.00	SAN	1.00	MDW	1.00	SEA	0.77	
PDX	1.00	PDX	1.00	PDX	1.00	HNL	1.00	DCA	1.00			
OAK	1.00	OAK	1.00	SJC	1.00	PDX	1.00	IAD	1.00			
SJC	1.00	SJC	1.00	OGG	1.00	OAK	1.00	SAN	1.00			
SMF	1.00	SMF	1.00	KOA	1.00	SJC	1.00	DAL	1.00			
OGG	1.00	OGG	1.00	LIH	1.00	OGG	1.00	HOU	1.00			
GUM	1.00	GUM	1.00	ITO	1.00	KOA	1.00	OAK	1.00			
KOA	1.00	KOA	1.00	ABE	1.00	LIH	1.00	SNA	1.00			
LIH	1.00	LIH	1.00	LAN	1.00	ITO	1.00	SJC	1.00			
ITO	1.00	ITO	1.00	SCE	1.00			ONT	1.00			
				LAW	1.00			BUR	1.00			
				ROW	1.00			LGB	1.00			
								HPN	1.00			
								TTN	1.00			
								ILG	1.00			
								CLD	1.00			

References

1 Hale, M. J., Hoskins, R. S. & Baker, M. G. Screening for Influenza A(H1N1)pdm09, Auckland International Airport, New Zealand. *Emerging Infectious Diseases* **18**, 866-868, doi:10.3201/eid1805.111080 (2012).

2 Center for Disease Control and Prevention (CDC). *H1N1 Flu (Swine Flu): Past Situation Updates*, <https://www.cdc.gov/h1n1flu/updates/> (2010).

3 Balcan, D. *et al.* Seasonal transmission potential and activity peaks of the new influenza A(H1N1): a Monte Carlo likelihood analysis based on human mobility. *BMC Medicine* **7**, 45, doi:10.1186/1741-7015-7-45 (2009).

4 Center for Disease Control and Prevention (CDC). Outbreak of Swine-origin influenza A (H1N1) virus infection—Mexico, March-April 2009. *Morb. Mortal. Wkly. Rep* **58**, 467-470 (2009).

5 World Health Organization (WHO). *First confirmed case of influenza A(H1N1) in Hong Kong (China)*, <http://www.wpro.who.int/mediacentre/news/2009/20090502/en/> (2009).
